# Chlorate of Potash in Phthisis—Notes of Three Cases Treated with Chlorate of Potash, in the Buffals General Hospital

**Published:** 1861-09

**Authors:** C. C. F. Gay

**Affiliations:** Attending Physician


### *Chlorate of Potash in Phthisis—Notes of three cases treated with Chlorate of Potash in the Buffalo General Hospital.* By C. C. F. Gay, M. D., Attending Physician. Reported by J. A. Peters, Medical Student.

---

Much discussion has been elicited lately by Dr. Fountain's proposed treatment of pulmonary consumption with chlorate of potassa. The following notes of three cases, treated in the Buffalo General Hospital, are submitted, in the hope that they may be of some use in determining the value of the remedy. It may be objected by the friends of the treatment, and with truth, that the trial has not been sufficient to disprove its value; but the standing of any remedy is only fixed by the comparison of its effects in many cases, in the hands of different practitioners, and it is to assist in this comparison that these cases are reported.

Case I.—Thomas Kay, laborer, English, æt. 21, unmarried, habits intemperate, came in June 17th; has had a cough nearly two months. Symptoms indicate well-marked tuberculosis, emaciation great. The treatment adopted at first was the usual course of anodynes, cod-liver oil, and tonics, under which he continued in much the same condition until July 16th, when nausea and diarrhœa made their appearance; these were treated by appropriate remedies.

21st. Night-sweats. ℞.—Acid. sulph. arom., gtt. x., every night.

From this time he continued improving until August 1st, when all treatment was stopped, except a prescription which he had been taking for some days past to correct nausea. He continued much the same—his appetite being small, and diarrhœa, with some nausea, being present until September 6th, when his tongue presented a very bad appearance, being covered with white aphthous patches, and ragged at the edges. On the 8th of September all previous treatment was stopped, and potass. chlor. was ordered, ℥ ss. to be drank, dissolved in water, in 24 hours.

9th. Whisky was resumed.

10th. Secretion of urine increased, much cough and expectoration of blood.

18th. Increased pot. chlor. to ℨ vj., daily.

22d. Tongue better; edges still sore.

26th. Vomits potash; and on the 29th the potash was discontinued. During this time, his diarrhœa had been increased rather than diminished, and was immediately checked on stopping the potash. Nausea had continued, and seemed to have direct reference to the potash since, although that medicine was well borne at first, after two or three weeks of its use he could scarcely bear the sight of it.

Case II.—John J. Cross, æt. 29, English, unmarried, formerly a butcher, but now an hostler, previous health good, taken five weeks since with chill and fever, followed by pain in side. In this case, the great emaciation and general appearance of the patient were such as to warrant the diagnosis of phthisis, which was further confirmed by physical exploration. Pulse 120 at the time, and has continued frequent ever since. He was ordered the following prescription on the 22d:

℞.—Potass. chlor. - - - ℥ ss.

Aquæ, - - - - - Oj.

To be drank in 24 hours.

24th. Could not sleep; appetite better; cough and expectoration slight.

25th. Has diarrhæa; gin ordered.

26th. Cough worse; diarrhœa continues.

28th. Diarrhœa still present. Condition generally anæmic. ℞. Sulph. quinæ.

October 3d. No better. Discontinued potash, which caused great nausea and lothing.

The diarrhœa in this case seemed to abate as soon as the pot. chlor. was stopped. The patient took the remedy well at first, and seemed to like it, but soon "loathed even the sight of it," as he said, and could not take it.

In the third case the remedy was ordered—the general symptoms seeming to warrant its use, although there were no strongly marked indications of tuberculosis. It is merely mentioned as illustrating the same effect which seemed to proceed, in the other cases, from the use of this remedy, viz: nausea and diarrhœa.

In neither of the above cases was any good effect perceptible; but, on the contrary, the appetite was diminished, the kiarrhœa and anæmia exacerbated, and nausea produced or increased. At first the patients were pleased to have a simple pleasant drink ordered, but this soon gave way to an intense disgust, and inability to swallow the medicine.

Whatever efficacy the remedy had in the hands of Dr. Fountaine, it has served no good purpose, and its use has been abandoned in this Hospital.—*Am. Med. Monthly.*

